# Combined Short-Chain Fatty Acids Induce an Anti-Inflammatory and Anti-Chemotactic Secretory Profile from 3T3-L1 Adipocytes in Normoxic and Hypoxic Environmental Conditions

**DOI:** 10.3390/ijms27156583

**Published:** 2026-07-24

**Authors:** Ala Alzubi, Hannah X. Glowacki, Kelsey Van, Clara E. Cho, Jennifer M. Monk

**Affiliations:** Department of Human Health Sciences, University of Guelph, Guelph, ON N1G 2W1, Canada; aalzubi@uoguelph.ca (A.A.); hglowack@uoguelph.ca (H.X.G.); kvan@uoguelph.ca (K.V.); claracho@uoguelph.ca (C.E.C.)

**Keywords:** short-chain fatty acids, acetate, propionate, butyrate, adipocytes, hypoxia, adipokines, inflammation, lipopolysaccharide, chemokines

## Abstract

Short-chain fatty acids (SCFAs), acetate, propionate, and butyrate, are typically produced in a 3:1:1 ratio, respectively, via microbial fermentation of non-digestible carbohydrates, and to a lesser degree, from undigested protein. The effects of individual SCFAs on adipocyte function have been described; however, the effects of SCFAs in combination on adipocyte function remain unknown. Mature 3T3-L1 adipocytes were treated with a 1 mM total dose of acetate, propionate, and butyrate combined in a 3:1:1 ratio, respectively, for 24 h ± lipopolysaccharide (LPS, 10 ng/mL) under both normoxic and hypoxic (via the addition of 100 µM cobalt chloride) environmental conditions. In both normoxic and hypoxic LPS-stimulated conditions, SCFAs increased the secretion of adiponectin and reduced the secretion of resistin, interleukin (IL)-6, monocyte chemoattractant protein (MCP)-1/C-C motif chemokine ligand (CCL)2, and RANTES/CCL5, in addition to reducing intracellular protein levels of activated (i.e., the ratio of phosphorylated-to-total) nuclear factor kappa-light-chain-enhancer of activated B cells (NFκB) p65 and signal transducer and activator of transcription 3 (STAT3) (*p* < 0.05). Additionally, SCFA treatment reduced leptin secretion only in LPS-stimulated normoxic environmental conditions compared to control (*p* < 0.05). In normoxic conditions, SCFA + LPS increased mRNA expression of genes involved in fat storage), fatty acid recycling, and lipolysis, whereas in hypoxic conditions, SCFA + LPS decreased mRNA expression of genes involved in fat storage and triglyceride synthesis (*p* < 0.05), indicating different effects of SCFAs on adipocyte metabolic function depending on hypoxia status. Collectively, combined SCFAs in a 3:1:1 ratio beneficially modify the adipocyte adipokine secretory profile under both normoxic and hypoxic environmental conditions.

## 1. Introduction

Changes in adipocyte function within obese adipose tissue, which include an increased proinflammatory adipokine secretory profile (comprised of adipose tissue-derived hormones, cytokines, and chemokines) that subsequently contributes to metabolic dysfunction locally within the adipose tissue, and upon release into the circulation, these adipokines contribute to systemic low-grade chronic inflammation (i.e., meta-inflammation) [1,2,3,4,5]. These inflammatory signals contribute to the development of obesity-associated metabolic dysfunction, which includes dyslipidemia and insulin resistance [1,2,3,4,5]. Furthermore, obese hypoxic adipose tissue can further exacerbate inflammatory adipokine secretion compared to normoxic adipose tissue environmental conditions [3,6,7,8,9,10]. Therefore, strategies that attenuate adipocyte dysfunction and reduce the secretion of inflammatory adipokines can reduce the severity of adipocyte dysfunction and/or the severity of the obese phenotype. Changes in microbiome composition and function are also associated with obesity [5,11], which can include the capacity to produce short-chain fatty acids (SCFAs) from microbial fermentation of non-digestible or microbially accessible carbohydrates [12,13]. Additionally, SCFAs can also be produced, albeit in lower concentrations, from the fermentation of undigested protein [14,15,16,17,18]. The most common SCFAs produced are acetate (or acetic acid), propionate (or propionic acid), and butyrate (butyric acid), typically in a 3:1:1 molar ratio that accounts for 90–95% of colonic SCFA levels [19,20,21,22,23], wherein the remainder of total colonic SCFA levels is attributable to longer carbon chain structures [24]. SCFA production is influenced by multiple factors, including (i) the microbiome composition (i.e., abundance of SCFA-producing microbial species), (ii) the amount and type(s) of non-digestible carbohydrates consumed in the diet (i.e., SCFA precursors including, but not limited to, soluble fibers, resistant starches, oligosaccharides, etc.), (iii) the relative fermentability of each type of non-digestible carbohydrate (i.e., fast versus slowly fermentable), and (iv) the intestinal SCFA absorptive capacity [14,25,26,27,28,29,30].

Importantly, SCFAs can cross the gastrointestinal epithelial barrier, and despite lower circulating SCFA concentrations compared to fecal or gastrointestinal luminal concentrations [19,31,32,33,34,35,36,37,38], once in the blood, SCFAs can modulate metabolic function in extra-intestinal tissues, including adipose tissue, liver, pancreas, and skeletal muscle [14,39,40,41,42]. Within tissues, SCFAs exert their effects primarily by binding to G protein-coupled receptors (GPRs), mainly GPR41, GPR43, and GPR109a, wherein acetate, propionate, and butyrate exert different receptor binding affinities [43,44,45]. SCFAs are frequently presumed to exert similar biological effects; however, studies have shown that SCFAs can exert individual effects and have some common effects on insulin-stimulated glucose uptake and inflammatory mediator secretion in skeletal muscle cells (i.e., myokine secretion) [46,47] and adipocytes (i.e., adipokine secretion) [48,49,50]. Specifically in adipocytes, the effects of individual SCFAs on adipokine secretion were dose-dependent, and the anti-inflammatory and anti-chemotactic effects of each SCFA (acetate, propionate, butyrate, and valerate) differed between normoxic and hypoxic adipocyte environmental conditions [48,49,50]. Thus, the effectiveness of SCFAs to mitigate adipocyte or adipose tissue dysfunction can depend on the severity of the physiological challenge the cells encounter, wherein inflammation-associated metabolic dysfunction is exacerbated in hypoxic obese adipose tissue [3,6,7,8,9,10]. Although it is relevant to identify the effects of individual SCFAs, what remains unknown is the effect of SCFAs in combination in the physiologically relevant molar ratio that they are typically produced within the gastrointestinal tract [19,20,21,22,23]. Therefore, the purpose of this study was to determine the effects of SCFAs when combined in a 3:1:1 molar ratio of acetate to propionate to butyrate, respectively, on adipocyte inflammatory and metabolic function under both normoxic and hypoxic environmental conditions.

## 2. Results

### 2.1. SCFA + Lipopolysaccharide (LPS) Reduce Adipokine mRNA Expression and Protein Secretion in Normoxic Environmental Conditions

**Figure 1:**
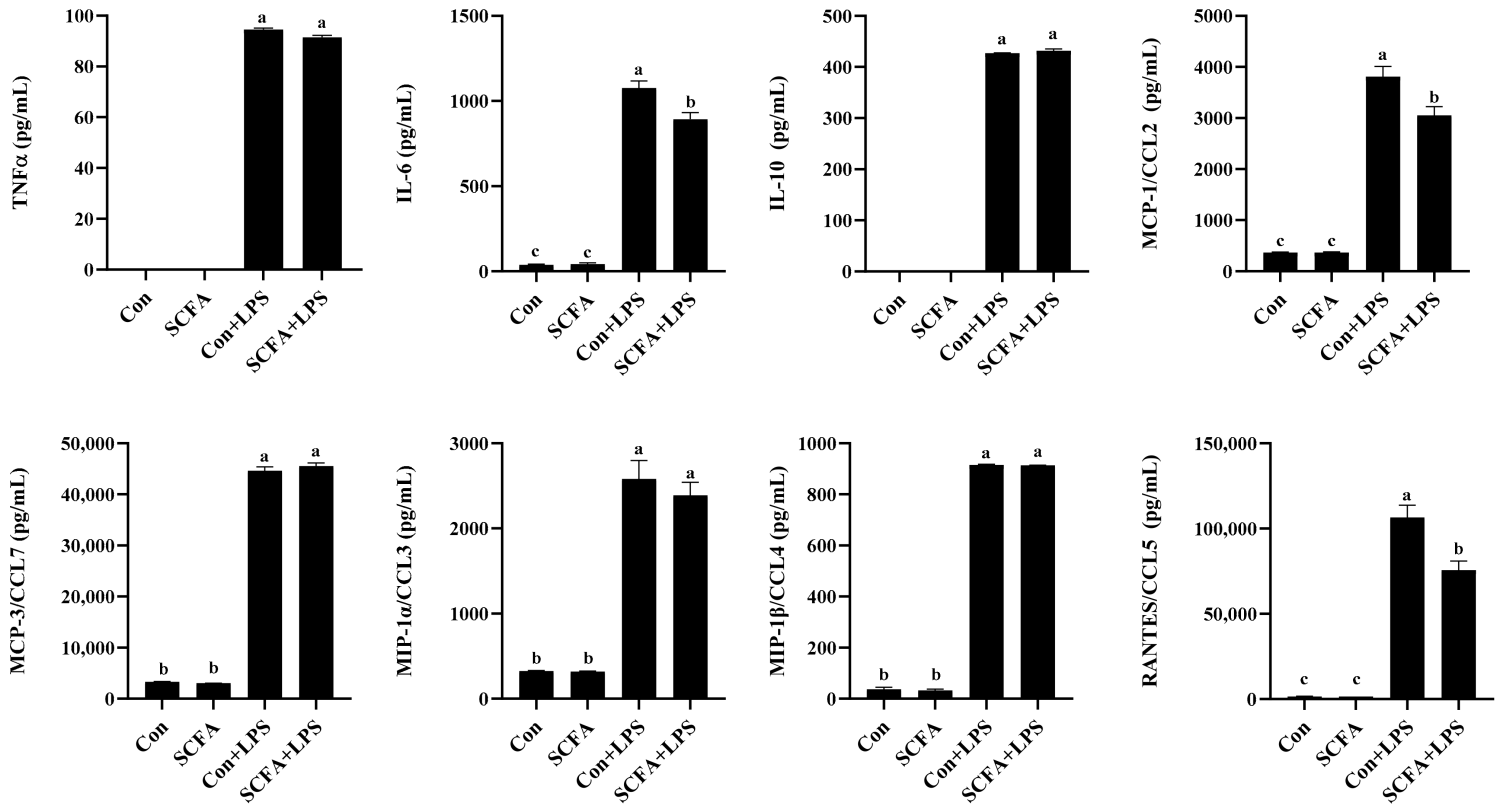
Secreted adipokine (cytokine and chemokine) protein levels from 3T3-L1 adipocytes treated with 1 mM total SCFAs (3:1:1 molar ratio of acetate–propionate–butyrate; SCFAs) or control (media; Con) under unstimulated (*n* = 6/experimental group: Con and SCFA) or stimulated conditions with the addition of 10 ng/mL LPS (Con + LPS and SCFA + LPS; *n* = 8/experimental group) for 24 h in normoxic environmental conditions. Data are presented as mean values ± SEM. Bars not sharing a lower-case letter are significantly different (*p* < 0.05).

**Figure 2:**
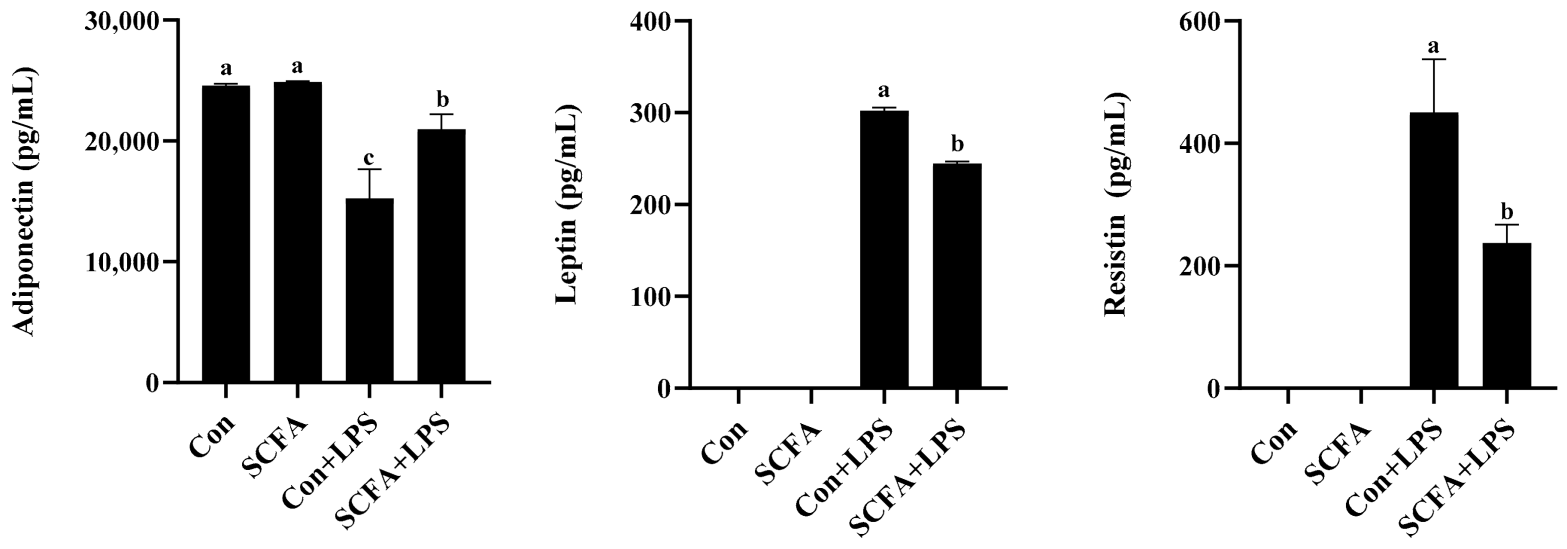
Secreted adipokine (hormone) protein levels from 3T3-L1 treated with 1 mM total SCFA (3:1:1 molar ratio of acetate–propionate–butyrate; SCFAs) or control (media; Con) under unstimulated (*n* = 6/experimental group: Con and SCFA) or stimulated conditions with the addition of 10 ng/mL LPS (Con + LPS and SCFA + LPS; *n* = 8/experimental group) for 24 h in normoxic environmental conditions. Data are presented as mean values ± SEM. Bars not sharing a lower-case letter are significantly different (*p* < 0.05).

Secreted adipokine protein levels from unstimulated (i.e., no LPS-stimulation, media alone with and without SCFA) and LPS-stimulated (i.e., LPS with and without SCFA) adipocyte cultures in normoxic environmental conditions are shown in Figure 1 and Figure 2.

Secreted adipokine levels were significantly lower in the non-LPS-stimulated conditions compared to LPS-stimulated for all adipokines (*p* < 0.05), with the exception of adiponectin, which exhibited higher levels in the unstimulated groups compared to LPS-stimulated (Figure 2). The secreted protein levels of transforming growth factor alpha (TNFα), interleukin (IL-)-10, leptin, and resistin were below the limit of detection in the non-LPS (i.e., unstimulated) Con and SCFA-treated groups. There were no differences in the secreted protein levels of any adipokines between the unstimulated Con and SCFA-treated groups (*p* > 0.05). In LPS-stimulated adipocyte cultures, which more accurately reflects inflammatory stimulation of obese adipose tissue [51,52,53], the presence of SCFA + LPS reduced IL-6, monocyte chemoattractant protein (MCP)-1/C-C motif chemokine ligand (CCL)2, and regulated upon activation, normal T cell expressed and secreted (RANTES)/CCL5 compared to Con + LPS (*p* < 0.05; Figure 1). There was no difference between the Con + LPS and SCFA + LPS groups in the secretion of TNFα, IL-10, MCP-3/CCL7, macrophage inflammatory protein (MIP)-1α/CCL3, or MIP-1β/CCL4. Conversely, adiponectin secretion was increased in the SCFA + LPS group compared to Con + LPS (*p* < 0.05; Figure 1). Secretion of the inflammatory adipokines leptin and resistin was reduced in the SCFA + LPS group compared to Con + LPS (*p* < 0.05; Figure 2).

**Figure 3:**
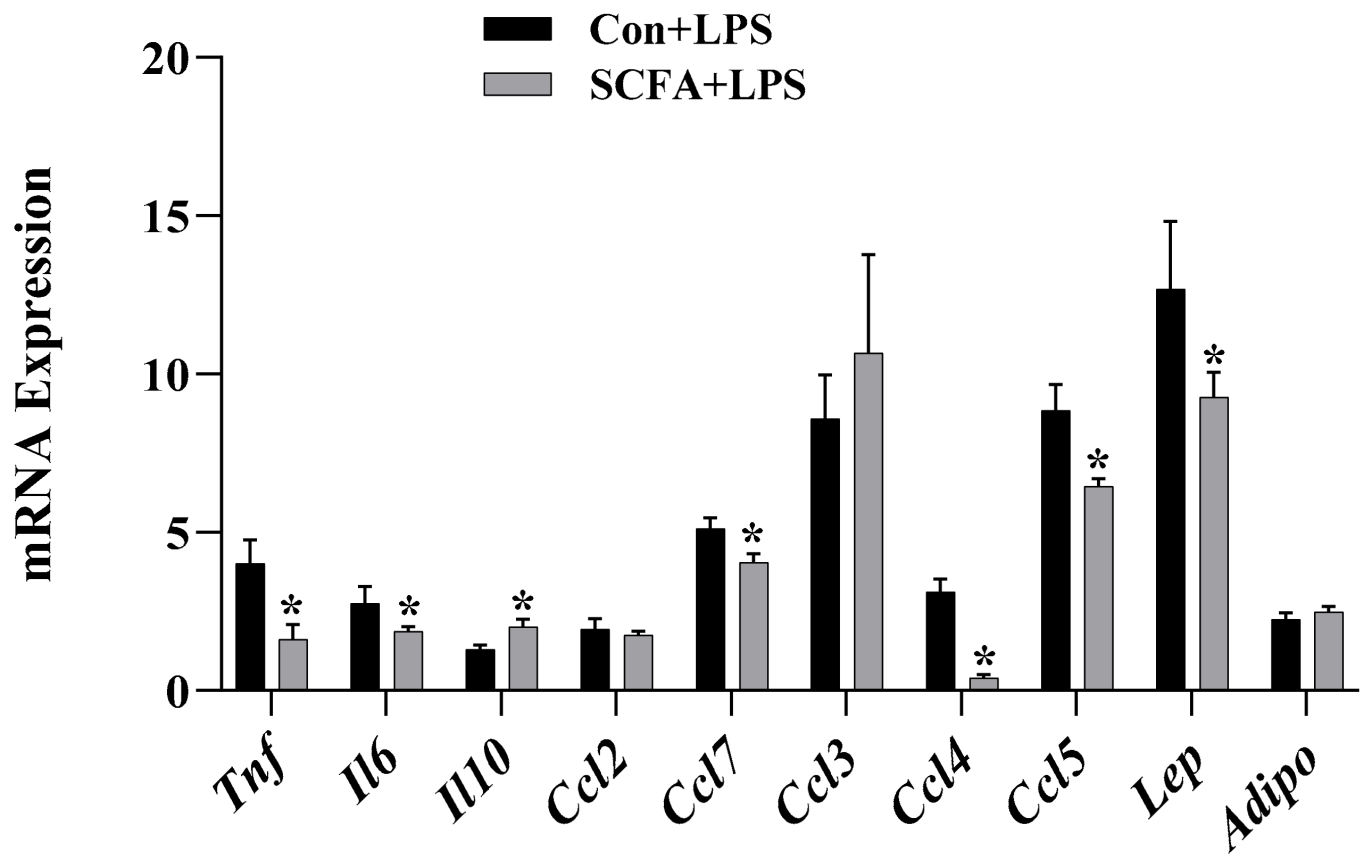
mRNA expression of adipokines in 3T3-L1 adipocytes stimulated with LPS (10 ng/mL) and treated with 1 mM total SCFA (3:1:1 molar ratio of acetate–propionate–butyrate; SCFA + LPS) or control (media; Con; Con + LPS) in normoxic environmental conditions (*n* = 8/experimental group) for 24 h. Data are presented as mean values ± SEM. Significant differences between experimental groups are indicated by an asterisk (*) (*p* < 0.05).

Specifically, in LPS-stimulated adipocyte cultures, SCFA reduced mRNA expression of the inflammatory cytokines *Tnfa* and *Il6*, along with the chemokines *Ccl7*, *Ccl4*, *Ccl5,* and the inflammatory adipokine leptin (*Lep*) compared to Con (*p* < 0.05; Figure 3). mRNA expression of the anti-inflammatory cytokine *Il10* was increased in the combined SCFA + LPS treatment group compared to Con + LPS (*p* < 0.05). There was no difference in mRNA expression of *Ccl2*, *Ccl3*, or adiponectin (*Adipo*) between the Con + LPS and SCFA + LPS groups (*p* > 0.05).

### 2.2. Transcription Factor Activation in Adipocytes Is Reduced by SCFA + LPS in Normoxic Environmental Conditions

**Figure 4:**
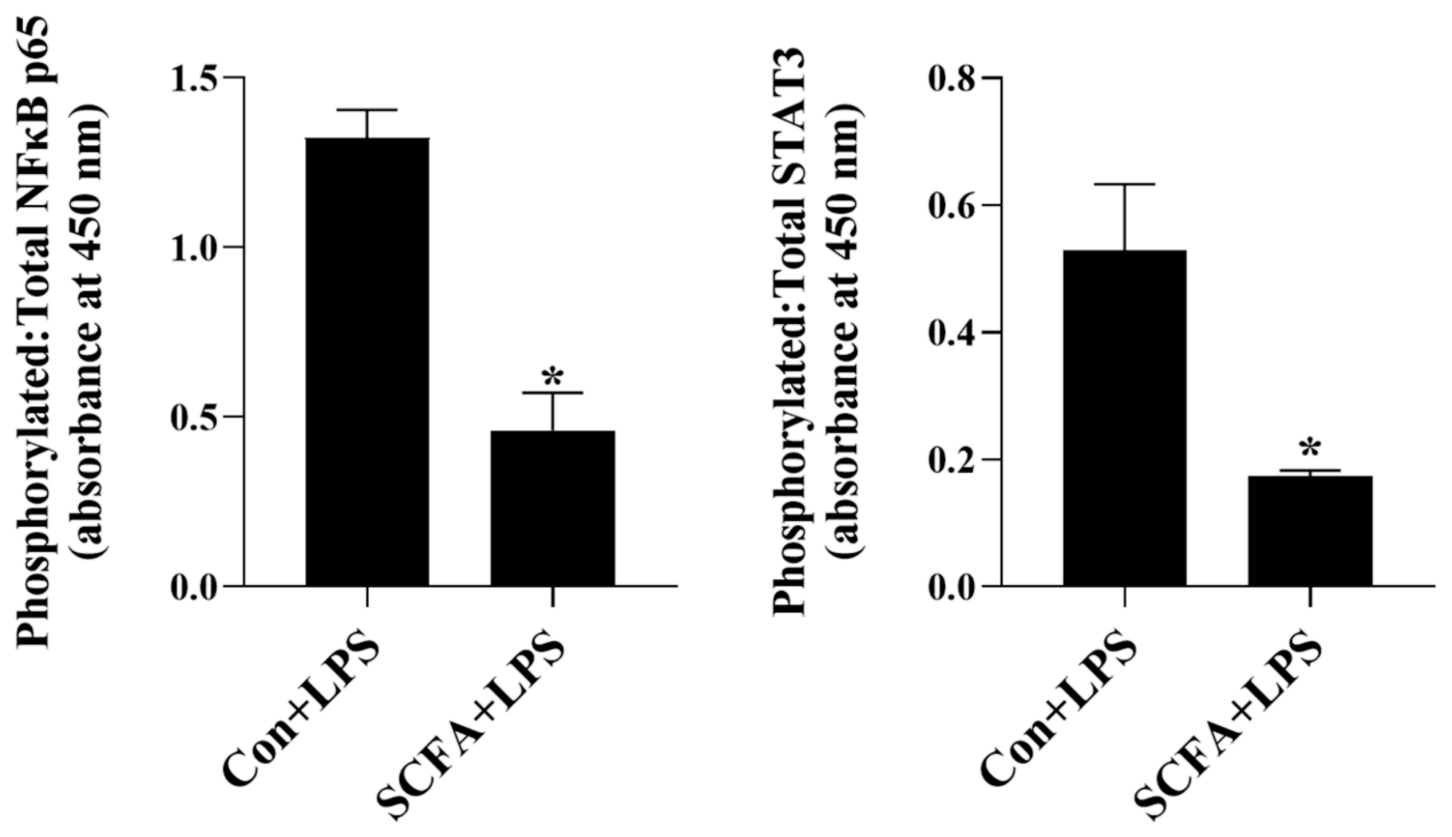
Activation status (i.e., ratio of phosphorylated–total) intracellular NFκB p65 and STAT3 protein levels from 3T3-L1 adipocytes stimulated with LPS (10 ng/mL) and treated with 1 mM total SCFA (3:1:1 molar ratio of acetate–propionate–butyrate; SCFA + LPS) or control (media; Con; Con + LPS) in normoxic environmental conditions (*n* = 8/experimental group) for 24 h. Data are presented as mean values ± SEM. Significant differences between experimental groups are indicated by an asterisk (*) (*p* < 0.05).

The activation status (i.e., the ratio of phosphorylated to total) intracellular protein expression of the transcription factors nuclear factor kappa-light-chain-enhancer of activated B cells (NFκB) p65 and signal transducer and activator of transcription 3 (STAT3) under LPS-stimulation conditions is shown in Figure 4. In the SCFA + LPS group, both NFκB p65 and STAT3 activation were reduced compared to Con + LPS (*p* < 0.05).

### 2.3. Gene Expression of Signaling Receptors and Metabolic Function Are Affected by SCFA + LPS in Normoxic Environmental Conditions

**Figure 5:**
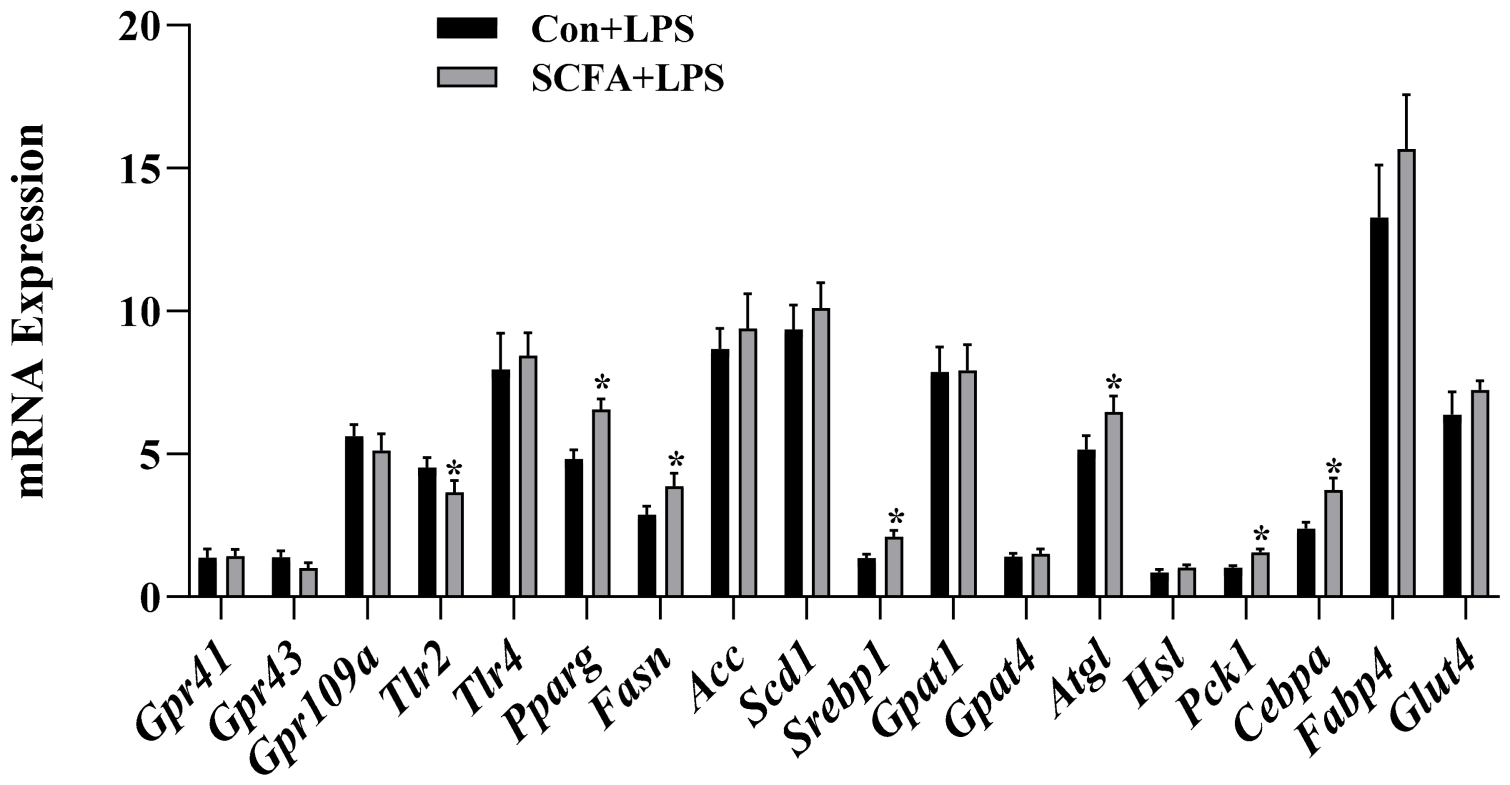
mRNA expression of signaling receptors and metabolism-related genes in 3T3-L1 adipocytes stimulated with LPS (10 ng/mL) and treated with 1 mM total SCFA (3:1:1 molar ratio of *acetate–propionate–butyrate*; SCFA + LPS) or control (media; Con; Con + LPS) in normoxic environmental conditions (*n* = 8/experimental group) for 24 h. Data are presented as mean values ± SEM. Significant differences between experimental groups are indicated by an asterisk (*) (*p* < 0.05).

GPR mRNA expression, namely *Gpr41*, *Gpr43*, and *Gpr109a*, did not differ between the SCFA + LPS and Con + LPS groups (Figure 5). The mRNA expression of the LPS signaling receptor toll-like receptor 2 (*Tlr2*) was decreased in SCFA + LPS-treated adipocyte cultures compared to Con + LPS (*p* < 0.05), whereas there was no difference in *Tlr4* mRNA expression between treatment groups (Figure 5).

In response to LPS stimulation under normoxic environmental conditions, mRNA expression of the lipogenesis-associated genes fatty acid synthase (*Fasn*), sterol regulatory element-binding protein 1 (*Srebp1*), and phosphoenolpyruvate carboxykinase 1 (*Pck1*) were all increased in the SCFA + LPS group compared to Con + LPS (*p* < 0.05; Figure 5). There was no difference in mRNA expression of acetyl-CoA carboxylase (*Acc*), stearoyl-CoA desaturase 1 (*Scd1*), glycerol-3-phosphate acyltransferase (*Gpat1*), and *Gpat4* between treatment groups (*p* > 0.05). Adipose triglyceride lipase (*Atgl*) mRNA expression was increased in the SCFA + LPS group (*p* < 0.05), whereas hormone-sensitive lipase (*Hsl*) expression did not differ between treatment groups. CCAAT/enhancer-binding protein alpha (*Cebpa*), and peroxisome proliferator-activated receptor-γ (*Pparg*) mRNA expression were also both increased in the SCFA + LPS group compared to Con + LPS (*p* < 0.05). There was no difference in mRNA expression of fatty acid binding protein 4 (*Fabp4*) and glucose transporter 4 (*Glut4*) between treatment groups.

### 2.4. In Hypoxic Environmental Conditions, SCFA + LPS Reduce Adipokine mRNA Expression and Protein

**Figure 6:**
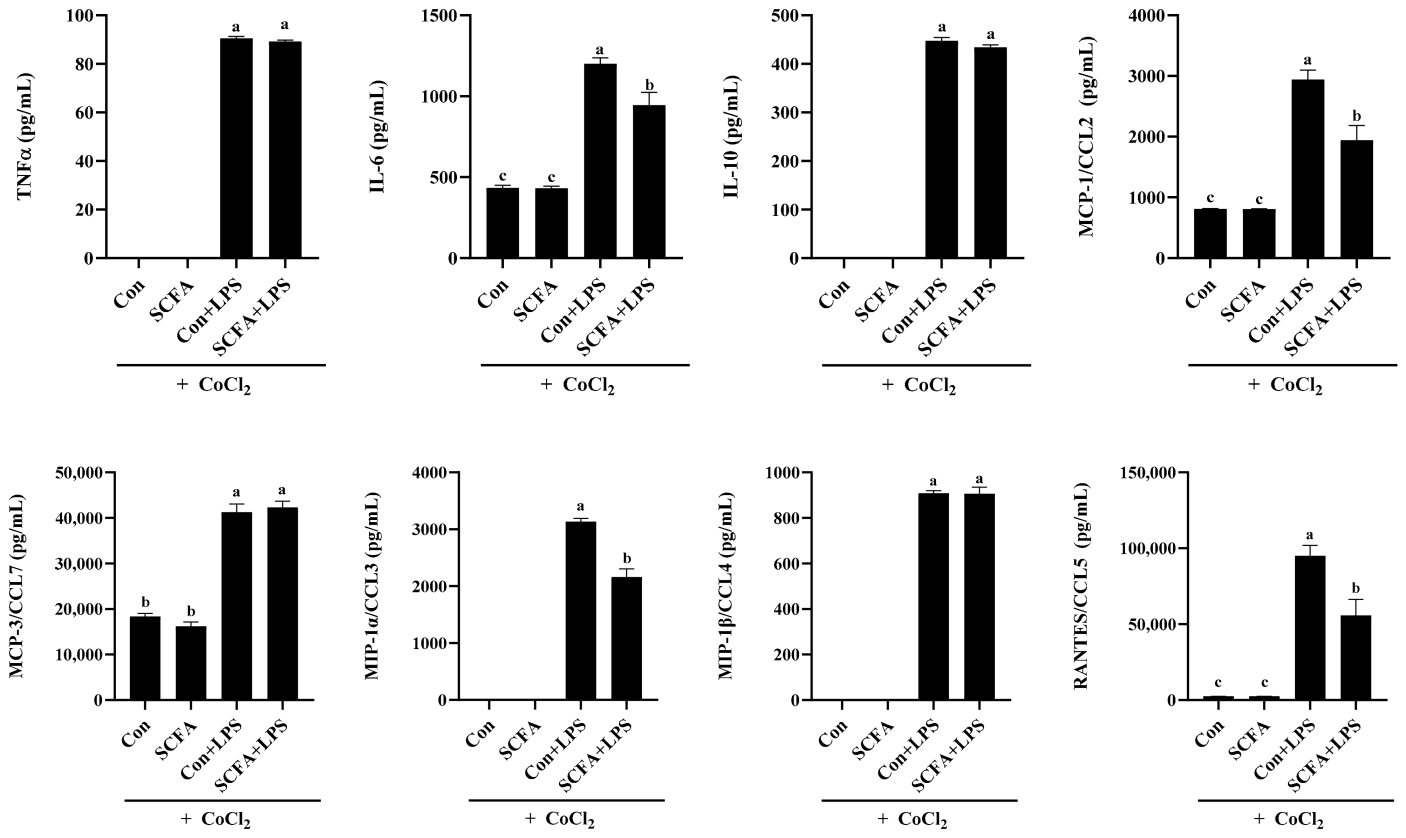
Secreted adipokine (cytokine and chemokine) protein levels from 3T3-L1 adipocytes treated without (media; control, Con) and with SCFA (1 mM of acetate–propionate–butyrate in a 3:1:1 molar ratio) that were also stimulated with LPS (10 ng/mL) in hypoxic environmental conditions induced via the addition of 100 µM of cobalt chloride (CoCl_2_) for 24 h (n = 8/experimental group: Con + LPS + CoCl_2_ and SCFA + LPS + CoCl_2_). Data are presented as mean values ± SEM. Bars not sharing a lower-case letter indicate significant differences between experimental groups (*p* < 0.05).

**Figure 7:**
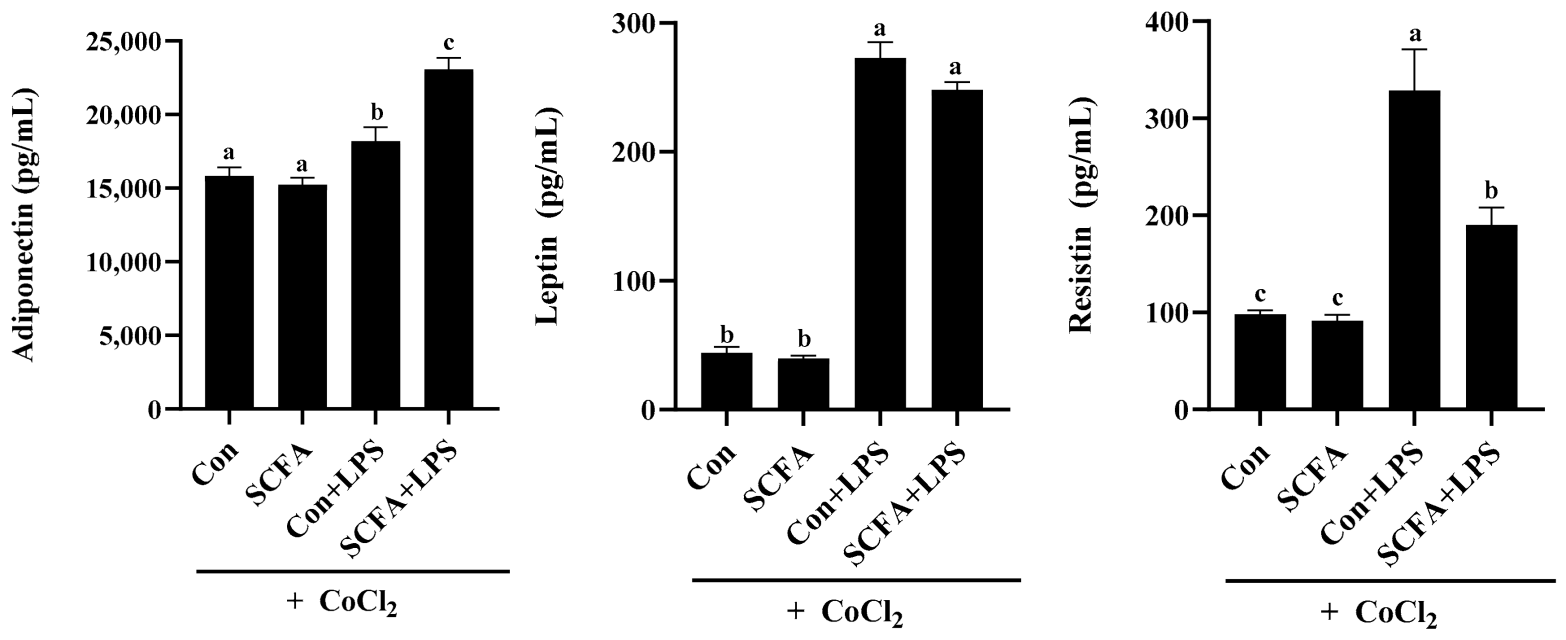
Secreted adipokine (hormone) protein levels from 3T3-L1 adipocytes treated without (media; control, Con) and with SCFA (1 mM of acetate–propionate–butyrate in a 3:1:1 molar ratio) that were also stimulated with LPS (10 ng/mL) in hypoxic environmental conditions induced via the addition of 100 µM of cobalt chloride (CoCl_2_) for 24 h (n = 8/experimental group: Con + LPS + CoCl_2_ and SCFA + LPS + CoCl_2_). Data are presented as mean values ± SEM. Bars not sharing a lower-case letter indicate significant differences between experimental groups (*p* < 0.05).

In hypoxic environmental conditions, the effect of the addition of SCFA to both non-LPS-stimulated (i.e., cobalt chloride (CoCl_2_) alone) and LPS-stimulated cultures of 3T3-L1 adipocytes (i.e., LPS + CoCl_2_) on adipokine secretion is shown in Figure 6 and Figure 7. There was no difference in any adipokine secreted protein level between the Con + CoCl_2_ and SCFA + CoCl_2_ groups in the unstimulated (i.e., no LPS) hypoxic conditions (*p* > 0.05), wherein TNFα, IL-10, MIP-1α/CCL3, and MIP-1β/CCL4 were not detected. The addition of the inflammatory stimulus LPS to hypoxic environmental conditions increased the secretion of all adipokines compared to unstimulated (i.e., no LPS) conditions (*p* < 0.05). Within the LPS-stimulated hypoxic conditions, SCFA reduced the secretion of IL-6, MCP-1/CCL2, MIP1α/CCL3, and RANTES/CCL5 compared to Con (*p* < 0.05; Figure 6). There was no difference in the secretion of TNFα, IL-10, MCP-3/CCL7, or MIP-1β/CCL4 between Con + LPS + CoCl_2_ and SCFA + LPS + CoCl_2_ groups. Additionally, secreted protein levels of adiponectin was increased (*p* < 0.05), and resistin secretion was decreased (*p* < 0.05) in the SCFA + LPS + CoCl_2_ group compared to Con + LPS + CoCl_2_ (Figure 7). There was no difference in leptin secretion between treatment groups (*p* > 0.05; Figure 7).

**Figure 8:**
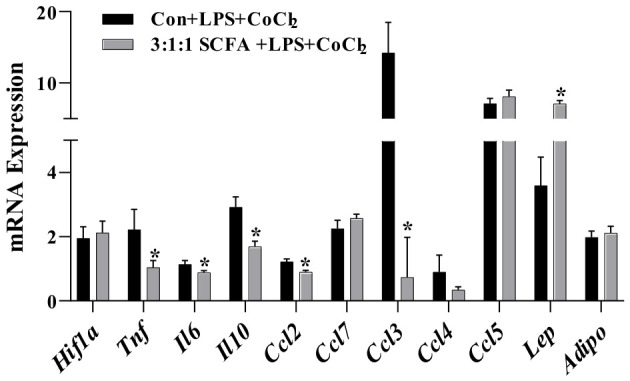
mRNA expression of adipokine and hypoxia-sensitive genes in 3T3-L1 adipocytes treated without (media; control, Con) and with SCFA (1 mM of acetate–propionate–butyrate in a 3:1:1 molar ratio) that were also stimulated with LPS (10 ng/mL) in hypoxic environmental conditions induced via the addition of 100 µM of cobalt chloride (CoCl_2_) for 24 h (*n* = 8/experimental group: Con + LPS + CoCl_2_ and SCFA + LPS + CoCl_2_). Data are presented as mean values ± SEM. Significant differences between experimental groups are indicated by an asterisk (*) (*p* < 0.05).

Within the LPS-stimulated hypoxic environmental condition, adipokine mRNA expression of the inflammatory cytokines *Tnf* and *Il6*, the anti-inflammatory cytokine *Il10*, and the chemokines *Ccl2* and *Ccl3* were all reduced in the SCFA + LPS + CoCl_2_ group compared to Con + LPS + CoCl_2_ (*p* < 0.05; Figure 8). Conversely, *Lep* mRNA expression was increased in the SCFA + LPS + CoCl_2_ group (*p* < 0.05), and there was no difference in *Ccl7*, *Ccl4*, *Ccl5*, or *Adipo* mRNA expression between experimental groups (*p* > 0.05). Finally, mRNA expression of *Hif1a*, a critical hypoxia-sensitive gene, did not differ between experimental groups (*p* > 0.05).

### 2.5. In Hypoxic Environmental Conditions, SCFA + LPS Reduce Transcription Factor Activation in Adipocytes

**Figure 9:**
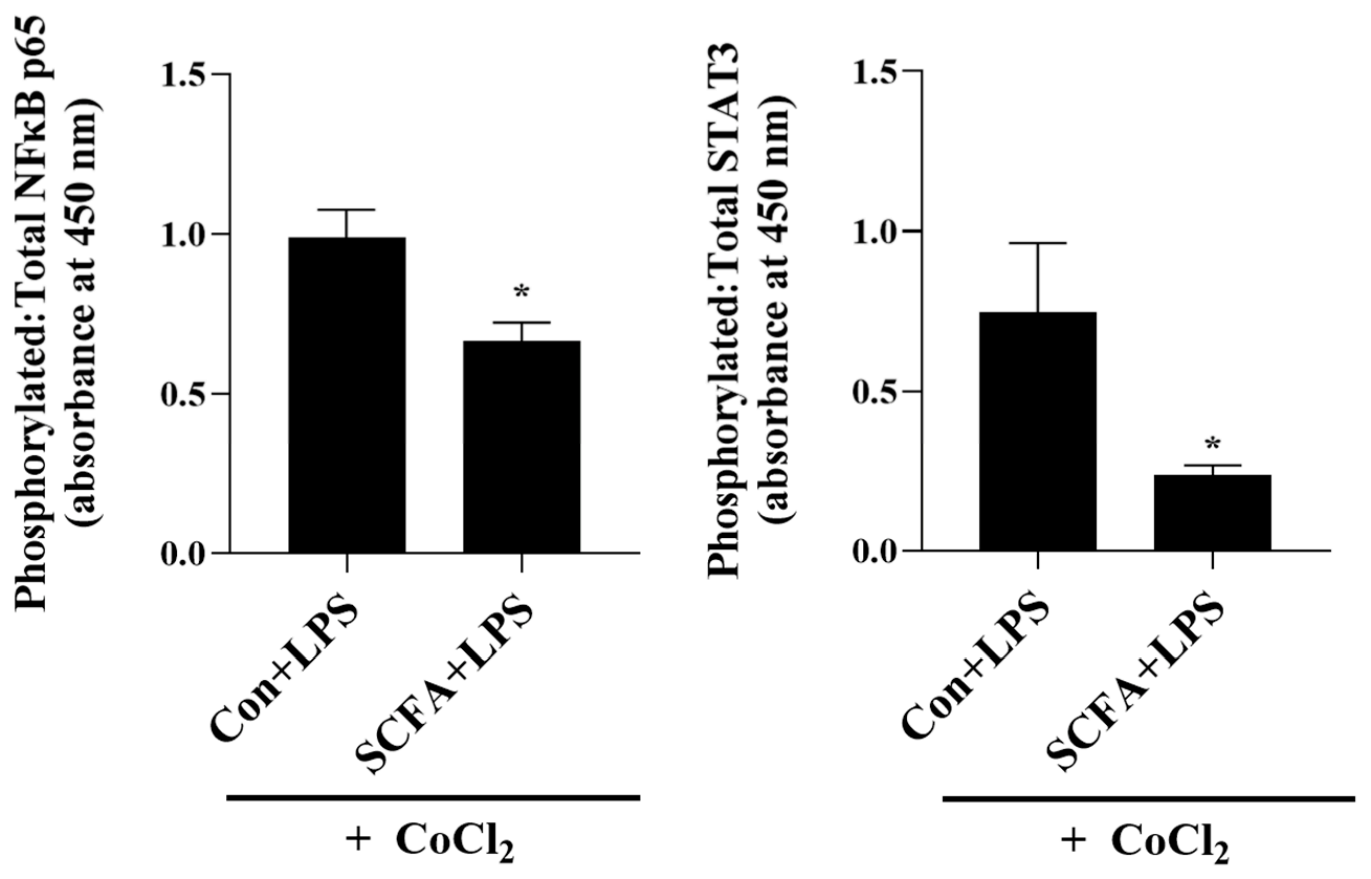
Activation status (i.e., ratio of phosphorylated: total) of NFκB p65 and STAT3 intracellular protein levels in 3T3-L1 adipocytes treated without (media; control, Con) and with SCFA (1 mM of acetate–propionate–butyrate in a 3:1:1 molar ratio) that were also stimulated with LPS (10 ng/mL) in hypoxic environmental conditions induced via the addition of 100 µM of cobalt chloride (CoCl_2_) for 24 h (*n* = 8/experimental group: Con + LPS + CoCl_2_ and SCFA + LPS + CoCl_2_). Data are presented as mean values ± SEM. Significant differences between experimental groups are indicated by an asterisk (*) (*p* < 0.05).

The activation status (i.e., the ratio of phosphorylated to total) intracellular protein expression of the transcription factors NFκB p65 and STAT3 under combined inflammatory and hypoxic (LPS + CoCl_2_) stimulation conditions is shown in Figure 9. In the SCFA + LPS + CoCl_2_ group, both NFκB p65 and STAT3 activation were reduced compared to Con + LPS + CoCl_2_ (*p* < 0.05).

### 2.6. In Hypoxic Environmental Conditions, mRNA Expression of Signaling Receptors and Metabolic Function Genes Are Affected by SCFA + LPS

**Figure 10:**
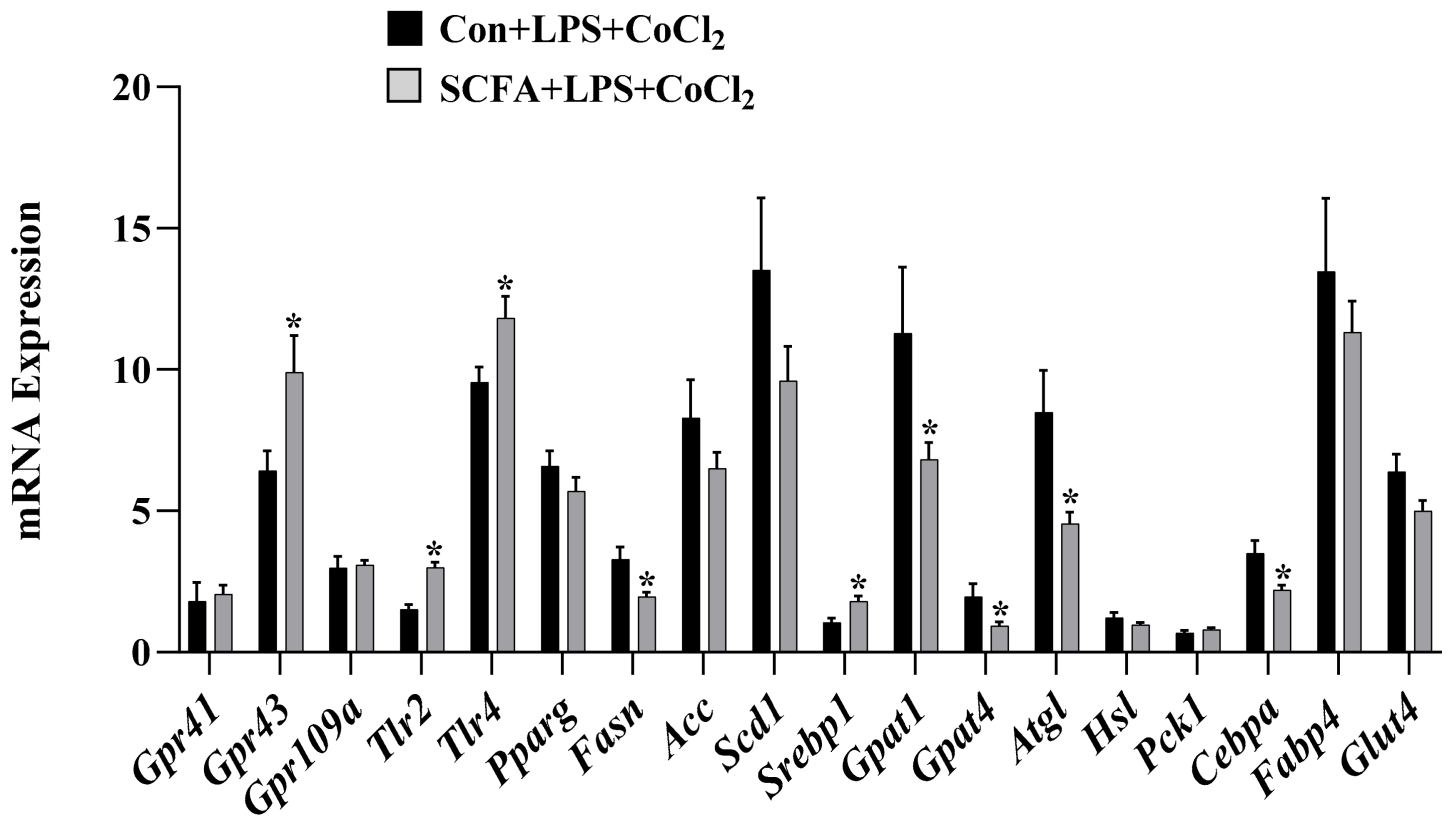
mRNA expression of signaling receptor and metabolism-related genes in 3T3-L1 adipocytes treated without (media; control, Con) and with SCFAs (1 mM of acetate–propionate–butyrate in a 3:1:1 molar ratio) that were also stimulated with LPS (10 ng/mL) in hypoxic environmental conditions induced via the addition of 100 µM of cobalt chloride (CoCl_2_) for 24 h (*n* = 8/experimental group: Con + LPS + CoCl_2_ and SCFA + LPS + CoCl_2_). Data are presented as mean values ± SEM. Significant differences between experimental groups are indicated by an asterisk (*) (*p* < 0.05).

mRNA expression of signaling receptor and metabolism-related genes was measured in LPS-stimulated adipocyte cultures in hypoxic environmental conditions. The SCFA signaling receptor, *Gpr43*, mRNA expression was increased in the SCFA + LPS + CoCl_2_ group compared to Con + LPS + CoCl_2_ (*p* < 0.05; Figure 10), whereas there was no difference in mRNA expression of the other SCFA signaling receptors, *Gpr41* or *Gpr109a*. Both *Tlr2* and *Tlr4* mRNA expression were increased in the SCFA + LPS + CoCl_2_ group compared to Con + LPS + CoCl_2_ (*p* < 0.05; Figure 10). Expression of select genes involved in metabolic function was reduced in the SCFA + LPS + CoCl_2_ group compared to Con + LPS + CoCl_2,_ including *Fasn*, *Gpat1*, *Gpat4*, *Agtl*, and *Cebpa*. Conversely, *Srebp1* mRNA expression was increased in the SCFA + LPS + CoCl_2_ group (*p* < 0.05), whereas mRNA expression of *Acc*, *Scd1*, *Hsl*, *Pck1*, *Fabp4*, *Glut4*, and *Pparg* did not differ between experimental groups.

## 3. Discussion

The current study utilized an adipocyte cell culture model to recapitulate obese adipocyte environmental conditions with a LPS concentration that mimics the circulating endotoxin levels reported in obese humans [53] and rodents in high-fat diet-induced obesity models [51,52]. Both normoxic and hypoxic environmental conditions were assessed, wherein adipokine secretion, metabolic and vascular dysfunction are more severe in hypoxic adipose tissue or adipocytes compared to normoxic conditions [3,6,7,8,9,10,54]. Therefore, under these two distinct environmental conditions, we investigated the effects of combined SCFA at a physiologically relevant 3:1:1 ratio of acetate, propionate, and butyrate, respectively, that reflects the production of 90–95% of colonic SCFA levels [19,20,21,22,23]. SCFA production and circulating levels in humans are variable and may not reflect a 3:1:1 ratio in the circulation [33,34,35,36,37,38], although ratios close to a 3:1:1 SCFA ratio have been reported in portal blood [55]. SCFA levels (within the gastrointestinal tract or in the blood) are influenced by the microbiome composition and activity, non-digestible carbohydrate dietary intake and relative fermentability, and intestinal SCFA absorptive capacity [14,25,26,27,28,29,30]. In this study, a 1 mM total concentration of SCFA was used, which has been used previously in functional studies and shown to be the most effective in dose-response studies that evaluated lower concentrations that more accurately reflect circulating concentrations [48,49,50,56,57,58,59,60,61]. The 3:1:1 SCFA ratio was selected as a starting point to evaluate the combined effects of SCFA, which is more physiologically relevant than individual SCFA, as these microbial metabolites are not produced in vivo in isolation but are produced concurrently, typically in a 3:1:1 ratio within the gastrointestinal tract. Importantly, this total SCFA concentration in the blood in a 3:1:1 ratio or some other ratio yet to be evaluated that effectively modulates adipocyte function could be achieved through encapsulated SCFA [62]. Thus, optimal SCFA delivery to the bloodstream through encapsulated supplements, which can increase circulating SCFA levels and expose extra-intestinal tissues to higher concentrations of SCFA than could be achieved through microbial fermentation of non-digestible dietary carbohydrates alone [62], represents the translational direction that the current study underpins. However, to adequately inform either SCFA supplementation recommendations or dietary intake recommendations for non-digestible carbohydrates with the intention of increasing the production of bioactive metabolites such as SCFA, it is necessary to identify the optimal SCFA concentration either as individual SCFA [48,49,50] and/or in combination (evaluated herein) that is required to exert beneficial extra-intestinal effects. Therefore, the extension of this work indicates that SCFA may serve as an adjuvant in attenuating the severity of inflammatory and/or metabolic dysfunction in obese adipose tissue.

Previous studies using in vitro models have identified SCFA-mediated effects on adipose metabolic function [42,49,61,63,64,65,66,67,68]; however, the effects of SCFAs on adipokines (gene expression or protein secretion) are seldom examined. The few studies evaluating SCFA effects on adipokine production have primarily focused only on leptin and adiponectin, and obtained inconsistent findings, in part due to variability in the SCFA concentration used (including the use of SCFA concentrations that exceed human circulating levels), only assessing select individual SCFA (e.g., propionate only versus inclusion of all SCFA), and/or using different adipocyte culture or adipose tissue explant models [57,60,63,64,69]. Comparisons of individual SCFAs on 3T3-L1 adipocyte function have shown that individual SCFAs do not exert the same effects on adipokine secretion, and the most pronounced anti-inflammatory and anti-chemotactic effect of SCFA is attributable to the longer carbon chain length fatty acids, such as butyrate [48,49]. In vivo, SCFAs are not produced in isolation, and instead are commonly produced in a 3:1:1 ratio of acetate–propionate–butyrate [19,20,21,22,23]. Therefore, this study evaluated the effects of combined SCFA on adipocyte function in normoxic and hypoxic environmental conditions, with categories of results summarized in Table 1. The results showed an anti-inflammatory and anti-chemotactic effects on adipocyte function that highlight the potential for SCFAs as an adjuvant for improving obesity-associated adipose tissue function.

In this study, combined SCFA in a 3:1:1 ratio (acetate–propionate–butyrate) exerted an anti-inflammatory and anti-chemotactic adipokine secretory profile under both normoxic and hypoxic conditions, wherein adipokine secretion is a critical adipocyte function that contributes to obesity-associated meta-inflammation and drives the development of systemic metabolic dysfunction, including dyslipidemia and insulin resistance [1,2,3,4,5]. Specifically, combined SCFA reduced secretion of critical adipokines such as IL-6, MCP-1/CCL2, RANTES/CCL5, and resistin, combined with increased secretion of adiponectin and a reduction in the activation of NFκB p65 and STAT3. Importantly, there were small differences in the secretory profile in response to SCFA, wherein leptin secretion was only reduced in normoxic environmental conditions, and secretion of the macrophage chemoattractant MIP-1α/CCL3 was reduced only in hypoxic conditions. The reduction in IL-6 secretion is relevant given its role in driving adipose tissue inflammation, influencing macrophage tissue infiltration, stimulating leptin secretion, and metabolic effects, including promoting lipolysis and impacting whole body metabolic regulation [70,71,72], wherein adipose tissue-derived IL-6 in obesity promotes insulin resistance and glucose intolerance [73,74,75]. The reduction in MCP-1/CCL2 secretion has functional relevance, as this chemokine is a critical signal for monocyte chemotaxis into adipose tissue, where the existing inflammatory microenvironment will promote the polarization of newly recruited monocytes into the M1 macrophage inflammatory functional phenotype, leading to increased inflammatory mediator production and insulin resistance [2,3,76,77,78]. Other chemokines that also contribute to immune cell recruitment to adipose tissue were reduced by SCFA in both normoxic and hypoxic environmental conditions, such as RANTES/CCL5, wherein circulating levels are elevated in individuals with impaired glucose tolerance [79]. Importantly, obese adipose tissue expression of RANTES is positively correlated with tissue macrophage levels [80] and increased T cell accumulation [81], thereby playing a key role in perpetuating immune cell recruitment and the ongoing production of inflammatory mediators that contribute to local adipose and systemic metabolic dysfunction and obesity co-morbidities [1,3]. Critical inflammatory adipokines, resistin and leptin, contribute to the severity of the obese phenotype. Resistin levels are increased in obesity [82,83] and have been shown to promote the inflammatory response through the activation of the transcription factor NFκB that drives inflammatory mediator expression, thereby inducing the secretion of multiple inflammatory cytokines and chemokines, including, but not limited to, MCP-1/CCL2, TNFα, and IL-6 [84]. In addition to metabolic influences of leptin on lipid metabolism and adipogenesis, leptin has also been shown to stimulate adipocytes and multiple immune cell types residing in adipose tissue to secrete inflammatory mediators, such as TNFα and IL-6 (amongst others) through signaling pathways leading to the activation of the transcription factors STAT3 and NFκB, ultimately creating a feedback loop that perpetuates chronic inflammation [85,86,87,88,89]. As evidence of the ability of SCFA to attenuate the adipocyte inflammation feedback loop, adipocyte expression of two critical transcription factors, NFκB p65 and STAT3, that upregulate the expression of many inflammatory adipokines [90], was reduced by SCFAs in both normoxic and hypoxic LPS-stimulated conditions. Furthermore, highlighting other transcription factors that change in response to SCFA, *Pparg* mRNA expression was reduced by SCFA + LPS in normoxic environmental conditions. PPARγ influences not only fatty acid metabolism and triglyceride storage, but also exerts beneficial effects in reducing the expression of inflammatory adipokines [91]. In contrast to the reduction in the inflammatory adipokine secretion, adiponectin secretion was increased by SCFA, which is functionally relevant as both an anti-inflammatory and insulin-sensitizing adipokine [92]. Typically, in obese adipocytes, adiponectin secretion is reduced, and the beneficial effects are lost [93,94]. Retention of adiponectin secretion with SCFAs represents an important adjuvant mechanism of action to attenuate adipocyte dysfunction.

Compared to normoxic obese adipose tissue, hypoxic obese adipose tissue is characterized by a more severe inflammatory phenotype and more severe metabolic dysfunction [3,8,95]. Obese adipocytes have been shown to increase oxygen consumption [96], which accounts for the approximated 40% decrease in interstitial oxygen tension in obesity and the decreased functional capillary density in obese adipose tissue [96]. Hypoxic conditions and inflammatory mediators such as IL-6 can perpetuate adipose tissue dysfunction, resulting in increased adipocyte expression of the transcription factor HIF-1α, which triggers the following cascade of events, including the activation of NFκB and STAT3, the subsequent production of more inflammatory adipokines, the increased immune cell chemotaxis and macrophage adipose tissue accumulation, and finally glucose intolerance and insulin resistance [3,95]. Therefore, the combined inflammatory and hypoxic (LPS + CoCl_2_) stimulation condition reflects a cell culture model of a more severe obese adipocyte phenotype [97,98]. In the current study, *Hif1a* mRNA expression did not differ between the Con and SCFA treatment groups, indicating a similar responsiveness to the environmental hypoxic condition; however, the production of inflammatory adipokines was reduced by SCFA + LPS + CoCl_2_. The use of chemically induced hypoxia provides an alternative approach when the equipment necessary for inducing environmental hypoxia conditions is not available, as CoCl_2_ functions to induce expression of HIF-1α and stabilize protein levels by preventing degradation, recapitulating the more severe hypoxic 1% oxygen environmental conditions [99]. Environmental and chemical hypoxia experimental approaches exert both overlapping and some distinct outcomes that may be cell type dependent [99]. The metabolic response to both hypoxia models is similar, although the activation of other signaling pathways has been shown to differ between hypoxia models, wherein the concentration of CoCl_2_ used, duration of hypoxia intervention, and cell type are variable across studies, as reviewed [99]. Future studies should evaluate the effects of SCFA in environmental hypoxia conditions wherein the severity of the hypoxia phenotype can be modified by the level of oxygen present, which can reproduce both the mild and severe obese adipose tissue hypoxic phenotypes.

Both TLR2 and TLR4 expression are upregulated in the adipose tissue from individuals with overweight or obesity [100], and LPS signaling through these receptors leads to the production of inflammatory mediators and ultimately insulin resistance [101]. In normoxic environmental conditions, SCFA + LPS reduced *Tlr2* mRNA expression, indicating that SCFA may reduce adipocyte responsiveness to inflammatory signaling, although *Tlr4* mRNA expression was unaffected. The redundant LPS-mediated signaling pathway utilizing TLR4 would be expected to remain intact. Interestingly, in hypoxic environmental conditions, both *Tlr2* and *Tlr4* RNA expression were increased by the SCFA + LPS + CoCl_2_ group; however, the inflammatory secretory profile and the expression of critical transcription factors at the termination of these signaling pathways that drive the expression of inflammatory adipokines were both reduced. SCFA signals through GPR, albeit with different binding affinities for each receptor (GPR41, GPR43, and GPR109a) [43,44,45]. Under normoxic conditions, there were no differences in GPR mRNA expression between SCFA + LPS and Con + LPS; however, under hypoxic conditions, *Gpr43* mRNA expression was increased by SCFA + LPS + CoCl_2_, indicating that adipocytes may be more responsive to GPR43-mediated signaling under hypoxic conditions. Previous studies using 3T3-L1 adipocytes have demonstrated that the metabolic effects of SCFA, including insulin responsiveness and glucose uptake, are mediated through GPR signaling [61]. Future studies may assess the specific signaling pathways utilized by SCFA under both normoxic and hypoxic environmental conditions to modulate adipocyte function through receptor antagonism studies; however, this is beyond the scope of the current study.

With respect to metabolic function, SCFAs have been shown to decrease lipolysis and increase triglyceride accumulation, along with increasing adipogenesis and glucose uptake [58,59,60,61,66,67,68,98], although the concentrations used in some studies exceed circulating SCFA levels in humans. Recently, we have shown no effect of individual SCFA (acetate, propionate, or butyrate) on adipocyte insulin-stimulated glucose uptake in the same inflammatory and normoxic environmental conditions utilized in this study [48]. In the current study, SCFA + LPS had no effect on *Glut4* mRNA expression in either normoxic or hypoxic environmental conditions. Propionate and butyrate have been shown to dose-dependently inhibit lipolysis and de novo lipogenesis, with the strongest effects observed at supraphysiological concentrations (10 mM) [102], whereas higher concentrations of acetate and propionate have been shown to promote lipid accumulation in adipocytes [103]. Lipid metabolism gene expression differed between normoxic and hypoxic inflammatory environmental conditions, specifically in normoxic conditions SCFA + LPS increased mRNA expression of genes involved in lipid storage (*Fasn*, *Srebp1*, *Cebpa*), fatty acid recycling (*Pck1*), and lipolysis (*Agtl*). These changes align with the effect of SCFA + LPS to reduce leptin secretion in normoxic conditions, which would normally function to inhibit lipolysis genes such as *Fasn* and *Srepb1* and stimulate lipolysis genes such as *Atgl* [85]. Conversely, in inflammatory hypoxic environmental conditions, lipid metabolism gene expression was decreased by SCFA, including *Fasn*, *Gpat1*, *Gpat4*, *Atgl*, and *Cebpa*, whereas *Srebp1* expression was increased, indicating differential effects of combined SCFA on metabolic function depending on hypoxia status. Our results suggest that despite consistent beneficial effects of combined SCFA on the adipokine secretory profile, the metabolic implications may differ depending on the hypoxia status of the adipose tissue.

When SCFA are combined in a 3:1:1 molar ratio at a concentration achievable through supplementation (alone or in combination with endogenous SCFA production from dietary precursors) [62] and using an adipocyte model that recapitulates the critical features of a normoxic or hypoxic obese adipose tissue microenvironment [48,49,51,52,53], we show that SCFA can attenuate the inflammatory adipokine secretory profile and influence metabolic function. Therefore, SCFA represent a microbial-derived metabolite from dietary precursors that can improve obese adipose tissue function, highlighting the critical connection of the gut-adipose communication axis. As proof-of-concept, dietary interventions with non-digestible carbohydrates have been shown to improve elements of the obese phenotype, including reducing weight gain [104,105,106,107,108,109,110,111,112,113,114,115,116,117,118,119], reducing adipose tissue mass [105,106,115,116,117,119,120,121,122], and improving metabolic outcomes, including dyslipidemia, blood glucose regulation, and/or insulin resistance [113,117,119,123,124,125,126,127,128]. In these studies, SCFA are the likely bioactive metabolite that underlies these beneficial extra-intestinal effects, which would be co-produced and function as a group in vivo, as utilized in the current study. Future studies should consider the time course of the effects of SCFA on adipose tissue function, including their ability to modify the adipokine secretory profile over time during both the initiation and resolution of an inflammatory response. Additionally, SCFA antagonism studies with GPR inhibitors [129] or histone deacetylase inhibitors [59] represent important future directions to determine the mechanisms through which SCFA exert their effects. Understanding how SCFA, in an optimal combination of individual types of SCFA, can exert beneficial effects in adipose tissue, represents a potential adjuvant role for SCFA as part of a non-invasive dietary intervention to attenuate obese adipose tissue dysfunction (either through supplementation alone or in combination with increased dietary non-digestible carbohydrate intake). In this connection, previous studies have shown that potential for dietary non-digestible carbohydrate interventions to attenuate the severity of the obese phenotype, including adipokine production and metabolic dysfunction [117,118,119,130], wherein the effects are likely driven by the production of SCFA. Future studies that leverage the findings from this study may include primary adipose tissue cultures to expand the breadth of metabolic functions influenced by SCFA under both normoxic and hypoxic conditions, beyond the anti-inflammatory and anti-chemotactic effects on the adipocyte adipokine secretory profile.

## 4. Materials and Methods

### 4.1. Cell Culture and 3T3-L1 Differentiation

3T3-L1 murine pre-adipocytes (CL-173; American Type Culture Collection, Manassas, VA, USA) were cultured and maintained according to the manufacturer’s instructions. Cells were grown and passaged as described previously [48,49,131] in Dulbecco’s modified Eagle’s medium (DMEM; HyClone, Logan, UT, USA) supplemented with 4 mM L-glutamine, 4500 mg/L glucose, 10% (*v*/*v*) low endotoxin sterile-filtered fetal bovine serum (FBS; Millipore-Sigma, Oakville, ON, Canada) and 1% (*v*/*v*) penicillin-streptomycin (Fisher Scientific, Mississauga, ON, Canada), henceforth “basic media”. Pre-adipocytes (at passage number 5) were differentiated into mature adipocytes as described [49,131,132], in brief, first with basic media supplemented with 1 µM dexamethasone, 0.5 mM 3-isobutyl-1-methylxanthine, and 10 µg/mL insulin (all from Millipore-Sigma), and subsequently maintained with basic media supplemented with 10 µg/mL insulin. Culture media was changed every two days, and on day 8 post-differentiation, adipocytes were incubated for 12 h in serum-free DMEM containing only 1% (*v*/*v*) penicillin-streptomycin prior to the addition of the experimental treatments (described below).

### 4.2. Cell Culture Conditions: SCFA Does, Normoxic and Hypoxic Experimental Stimulation

Mature 3T3-L1 adipocyte cultures were treated for 24 h with basic media alone (control; Con) or with SCFA (basic media plus 1 mM total concentration of SCFA (Millipore-Sigma) dissolved directly in the culture media in a 3:1:1 ratio of acetate (0.6 mM), propionate (0.2 mM), and butyrate (0.2 mM), a physiologically relevant ratio that reflects gastrointestinal SCFA production [19,20,21,22,23]. The concentration of total SCFA (1 mM) has been used previously for SCFA functional studies [56,57,58,59,60,61] and was shown to have the strongest effect on adipocyte function in dose-response studies [48,50]. Although the 1 mM concentrations are modestly above the circulating SCFA levels measured in humans [34,35,36,37,38]. Higher doses can be achieved in the blood via encapsulated SCFA supplementation strategies [62].

During the 24 h period, adipocytes cultures from both Con and SCFA-treated groups were subjected to the following stimulation conditions: (i) unstimulated normoxic (basic media, normoxic environmental conditions: 37 °C and 5% CO_2_; *n* = 6/experimental group: Con (negative control) and SCFA), (ii) LPS stimulated under normoxic environmental conditions (10 ng/mL LPS from *Escherichia coli* 055:B5 (Millipore Sigma); *n* = 8/experimental group: Con + LPS and SCFA + LPS), (iii) unstimulated hypoxic conditions (basic media alone at 37 °C and 5% CO_2_ plus 100 µM of the hypoxia-mimetic compound cobalt chloride (CoCl_2_; Millipore-Sigma); *n* = 8/experimental group: Con + CoCl_2_ and SCFA + CoCl_2_), and (iv) LPS-stimulated hypoxic conditions (10 ng/mL LPS plus 100 µM CoCl_2_ at 37 °C and 5% CO_2_; *n* = 8/treatment group: Con + LPS + CoCl_2_ and SCFA + LPS + CoCl_2_). The dose of CoCl_2_ was used to mimic the effects of low (1%) oxygen tension between adipocytes over the 24 h period without affecting cell viability [49,97,98]. Cell viability in response to all treatment conditions was assessed using Trypan blue staining, and live cell counts ranged between 90–95% with no difference between control and SCFA-treated cells, and there was no difference in total cellular protein content measured in normoxic or hypoxic adipocyte cultures, as seen previously [49,98]. The dose of LPS utilized in these experiments recapitulates the circulating endotoxin levels reported in obese humans [53] and rodent high-fat-diet-induced obesity models [51,52]. After 24 h, culture supernatant was collected and stored at −80 °C to await secreted adipokine analysis, and cells were lysed using the lysis buffer from the RNA/Protein Purification Plus Kit (#48200; Norgen Biotek Corp., Thorold, ON, Canada). All lysed cell extracts were stored at −80 °C and then processed to isolate both RNA and cellular protein using the same kit, as per the manufacturer’s instructions.

### 4.3. Gene Expression Analysis

Isolated RNA was quantified using the Nanodrop UV spectrophotometer (Model ND-2000; Thermo-Fisher Scientific, Burlington, ON, Canada). Only RNA samples with ratios (A260/A230 and A260/A280) between 1.8 and 2.2 were used for cDNA synthesis, wherein 2 µg of cDNA was synthesized using the high-capacity reverse transcription kit (Applied Biosystems, Forest City, CA, USA). Real-time PCR was conducted using the CFX q-PCR system (Bio-Rad, Mississauga, ON, Canada), as previously described [133], and used the following cycling conditions: one denaturing cycle at 95 °C followed by 95 °C for 4 s and 55.9 °C for 30 s, followed by 40 °C for 4 s. Expected PCR product sizes ranged between 70 and 200 base pairs. The validated primer sequences that have been used previously and were designed using the Roche Universal Probe Library and Assay Design Center are provided in Table 2. Primer sequences. Relative gene expression was normalized to the housekeeping gene, ribosomal protein, large, P0 (*Rplp0*), and calculated using the 2^−ΔΔCT^ method.

### 4.4. Intracellular and Secreted Protein Analysis

STAT3 (phosphorylated STAT3 [Tyr705]–total STAT3) and NFκB p65 (phosphorylated NFκB p65 [Ser536]–total NFκB p65) intracellular protein expression was measured using an enzyme-linked immunosorbent assay (Thermo-Fisher Scientific) as described previously [48]. Secreted proteins (IL-6, IL-10, TNFα, MCP-1/CCL2, MCP-3/CCL7, MIP-1α/CCL3, MIP-1β/CCL4, and RANTES)/CCL5, leptin, resistin and adiponectin) were measured in culture supernatant using Bio-Plex kits and equipment (Bio-Rad), as described [48].

### 4.5. Statistical Analysis

All data are expressed as means ± SEM. Comparisons between unstimulated and LPS-stimulated groups (in either normoxic or hypoxic environmental conditions) were conducted using a two-way ANOVA (main effects: treatment (i.e., Con and SCFA-treated) and stimulus (i.e., unstimulated versus LPS-stimulated), followed by Tukey’s multiple comparison test for post hoc analysis. The Shapiro-Wilk test was used to test for normality. For comparisons between two groups (i.e., within LPS-stimulated Con and SCFA groups), data were analyzed using an unpaired *t*-test with Welch’s correction. The predetermined upper limit of probability for statistical significance was set at *p* ≤ 0.05. All analyses were conducted using GraphPad Prism, version 10 (GraphPad Software, Inc., La Jolla, CA, USA).

## Figures and Tables

**Table 1:** Summary of Experimental Findings.

	Effect of 3:1:1 SCFA Ratio + LPS in Normoxic Conditions	Effect of 3:1:1 SCFA Ratio + LPS in Hypoxic Conditions
Adipokine Gene Expression	↓ *Tnf*, *Il6*, *Ccl7*, *Ccl4*, *Ccl5*, *Lep*	↓ *Tnf*, *Il6*, *Il10*, *Ccl2*, *Ccl3*, *Lep*
Secreted Adipokines (hormones, cytokines, and chemokines)	↓ IL-6 ↓ MCP-1/CCL2 ↓ RANTES/CCL5 ↑ Adiponectin ↓ Leptin ↓ Resistin	↓ IL-6 ↓ MCP-1/CCL2 ↓ MIP-1α/CCL3 ↓ RANTES/CCL5 ↑ Adiponectin ↓ Resistin
Transcription Factor Activation (ratio of phosphorylated–total)	↓ NFκB p65 ↓ STAT3	↓ NFκB p65 ↓ STAT3
Receptors and Transcription Factor Gene Expression	↓ *Tlr2* ↑ *Pparg*	↑ *Gpr43*, *Tlr2*, *Tlr4*
Lipid Metabolism Gene Expression	↑ *Srebp1*, *Atgl*, *Pck1*, *Cebpa*	↓ *Fasn*, *Gpat4*, *Atgl*, *Cebpa* ↑ *Srebp1*

Arrows indicate statistically significant differences (*p* < 0.05) in the SCFA group from Control. ↑ indicates increased expression and ↓ indicates decreased expression.

**Table 2:** Primer Sequences.

Gene	Forward Primer (5′–3′)	Reverse Primer (5′–3′)	References
*Rplp0*	ACTGGTCTAGGACCCGAGAAG	TCCCACCTTGTCTCCAGTCT	[98,134,135]
*Tnf*	CATCTTCTCAAAATTCGAGTGACAA	TGGGAGTAGACAAGGTACAACCC	[98,134,135]
*Il6*	AACGATGATGCACTTGCAGA	GAGCATTGGAAATTGGGGTA	[98,134,135]
*Il10*	GGTTGCCAAGCCTTATCGGA	ACCTGCTCCACTGCCTTGCT	[98,134,135]
*Ccl2*	GCCTGCTGTTCACACAGTTGC	CAGGTGAGTGGGGCGTTA	[98,134,135]
*Ccl7*	GCTGCTTTCAGCATCCAAGTG	CCAGGGACACCGACTACTG	[136]
*Ccl3*	CAGCTTATAGGAGATGGAGCTATG	TCACTGACCTGGAACTGAATG	[137]
*Ccl4*	TTCCTGCTGTTTCTCTTACACCT	CTGTCTGCCTCTTTTGGTCA	[136]
*Ccl5*	TCCAATCTTGCAGTCGTGTTTG	TCTGGGTTGGCACACACTTG	[134,138]
*Lep*	CAGCTGCAAGGTGCAAGAAG	GATACCGACTGCGTGTGTGA	[98]
*Adipo*	CAGGCATCCCAGGACATC	TCTCACCCTTAGGACCAAGAAG	[98]
*Gpr41*	GTGACCATGGGGACAAGCTTC	CCCTGGCTGTAGGTTGCATT	[130,139]
*Gpr43*	GGCTTCTACAGCAGCATCTA	AAGCACACCAGGAAATTAAG	[130,139]
*Gpr109a*	ATGGCGAGGCATATCTGTGTAGCA	TCCTGCCTGAGCAGAACAAGATGA	[130,139]
*Tlr2*	GGGGCTTCACTTCTCTGCTT	AGCATCCTCTGAGATTTGACG	[98,135]
*Tlr4*	AGAAAATGCCAGGATGATGC	CTGATCCATGCATTGGTAGGT	[98,135,138]
*Pparg*	TGCTGTTATGGGTGAAACTCTG	CTGTGTCAACCATGGTAATTTCTT	[140,141]
*Fasn*	CCAAATCCAACATGGGACA	TGCTCCAGGGATAACAGCA	[140]
*Acc*	GCGTCGGGTAGATCCAGTT	CTCAGTGGGGCTTAGCTCTG	[140]
*Scd1*	GAGACCTGATACCTAACACTCTGTCA	GATGTGATGTTTTCTTCTAGACTTTCC	[140]
*Srebp1*	ATCGGCGCGGAAGCTGTCGGGGTAGCGTC	ACTGTCTTGGTTGTTGATGAGCTGGAGCAT	[140]
*Gpat1*	AGCAAGTCCTGCGCTATCAT	CTCGTGTGGGTGATTGTGAC	[142]
*Gpat4*	TGTCTGGTTTGAGCGTTCTG	TTCTGGGAAGATGAGGATGG	[143]
*Atgl*	TGACCATCTGCCTTCCAGA	TGTAGGTGGCGCAAGACA	[140]
*Hsl*	CACAAAGGCTGCTTCTACGG	GGAGAGAGTCTGCAGGAACG	[140]
*Pck1*	GGAGTACCCATTGAGGGTATCAT	GCTGAGGGCTTCATAGACAAG	[144]
*Cebpa*	TCTCCCCTAGTTGTCCAAGG	GGGTGGGGAAGCCTAAGTC	[140,141]
*Fabp4*	TCGACCACAATAAAGAGAAAACG	CTTGTGGAAGTCACGCCTTT	[140]
*Glut4*	GACGGACACTCCATCTGTTG	GCCACGATGGAGACATAGC	[145]
*Hif1a*	AGGCTGGGAAAAGTTAGGAGTG	GGCAGCGATGACACAGAAAC	[49,98]

## Data Availability

The original contributions presented in this study are included in the article. Further inquiries can be directed to the corresponding author.

## Abbreviations

The following abbreviations are used in this manuscript:

LPS	Lipopolysaccharide
CoCl_2_	Cobalt chloride
SCFA	Short-chain fatty acids
GPR	G-protein coupled receptor
TNFα	Tumor necrosis factor alpha
IL	Interleukin
MCP	Macrophage chemoattractant protein
MIP	Macrophage inflammatory protein
RANTES	Regulated upon Activation, Normal T cell Expressed and Secreted
CCL	C-C Motif Chemokine Ligand
HIF-1α	Hypoxia-inducible factor-1 alpha
NFκB	Nuclear factor kappa-light-chain-enhancer of activated B cells
STAT3	Signal Transducer and Activator of Transcription 3
PPARγ	Peroxisome proliferator-activated receptor-γ
TLR	Toll-like receptor
Rplp0	ribosomal protein, large, P0
Cebpa	CCAAT/enhancer-binding protein alpha
Fasn	Fatty Acid Synthase
Acc	Acetyl-CoA Carboxylase
Scd1	Stearoyl-CoA Desaturase 1
Srebp1	Sterol Regulatory Element-binding Protein 1
Gpat	Glycerol-3-phosphate Acyltransferases
Atgl	Adipose Triglyceride Lipase
Hsl	Hormone-Sensitive Lipase
Pck1	Phosphoenolpyruvate Carboxykinase 1
Fabp	Fatty acid binding proteins
Glut4	Glucose Transporter 4
